# A Plasma Proteomic Approach in Rett Syndrome: Classical versus Preserved Speech Variant

**DOI:** 10.1155/2013/438653

**Published:** 2013-12-23

**Authors:** Alessio Cortelazzo, Roberto Guerranti, Claudio De Felice, Cinzia Signorini, Silvia Leoncini, Alessandra Pecorelli, Claudia Landi, Luca Bini, Barbara Montomoli, Claudia Sticozzi, Lucia Ciccoli, Giuseppe Valacchi, Joussef Hayek

**Affiliations:** ^1^Department of Medical Biotechnologies, University of Siena, Via A. Moro 2, 53100 Siena, Italy; ^2^Child Neuropsychiatry Unit, University Hospital Azienda Ospedaliera Universitaria Senese (AOUS), Viale M. Bracci 16, 53100 Siena, Italy; ^3^Neonatal Intensive Care Unit, University Hospital AOUS, Viale M. Bracci 16, 53100 Siena, Italy; ^4^Department of Molecular and Developmental Medicine, University of Siena, Via A. Moro 6, 53100 Siena, Italy; ^5^Department of Life Science, University of Siena, Via A. Moro 2, 53100 Siena, Italy; ^6^Department of Life Sciences and Biotechnology, University of Ferrara, Via Borsari 46, 44100 Ferrara, Italy; ^7^Department of Food and Nutrition, Kyung Hee University, 1 Hoegi-dong, Dongdaemun-gu, Seoul 130-701, Republic of Korea

## Abstract

Rett syndrome (RTT) is a progressive neurodevelopmental disorder mainly caused by mutations in the gene encoding the methyl-CpG-binding protein 2 (MeCP2). Although over 200 mutations types have been identified so far, nine of which the most frequent ones. A wide phenotypical heterogeneity is a well-known feature of the disease, with different clinical presentations, including the classical form and the preserved speech variant (PSV). Aim of the study was to unveil possible relationships between plasma proteome and phenotypic expression in two cases of familial RTT represented by two pairs of sisters, harbor the same *MECP2* gene mutation while being dramatically discrepant in phenotype, that is, classical RTT versus PSV. Plasma proteome was analysed by 2-DE/MALDI-TOF MS. A significant overexpression of six proteins in the classical sisters was detected as compared to the PSV siblings. A total of five out of six (i.e., 83.3%) of the overexpressed proteins were well-known acute phase response (APR) proteins, including alpha-1-microglobulin, haptoglobin, fibrinogen beta chain, alpha-1-antitrypsin, and complement C3. Therefore, the examined RTT siblings pairs proved to be an important benchmark model to test the molecular basis of phenotypical expression variability and to identify potential therapeutic targets of the disease.

## 1. Introduction

Rett syndrome (RTT; OMIM no. 312750), with a frequency of ~1 : 10000–1 : 15000 females, is a severe and complex neurodevelopmental disorder, as well as the second most common cause of severe mental retardation in the female gender [1]. RTT presents in about 74% of cases in a classical form (typical presentation); after 6–18 months of an apparently normal development girls lose their acquired cognitive, social, and motor skills in a typical 4-stage neurological regression. A wide phenotypical heterogeneity is a well-known feature of the disease, which includes at least four major different clinical presentations, that is, classical, preserved speech (PSV), early seizure (ESV), and congenital variants [2]. Studies have implicated *de novo* mutations of the X-linked methyl-CpG-binding protein 2 (*MECP2*) gene (OMIM∗300005) in the majority of the RTT cases, while mutations in cyclin-dependent kinase-like 5 (*CDKL5*) and forkhead box G1 (*FOXG1*) have been more rarely reported [3–5]. Typical RTT has been described worldwide, whereas PSV is more rarely reported. Girls affected by PSV have been often misreported with various diagnoses ranging from autism to mental retardation [6, 7].

While the available RTT literature is mainly focusing on the molecular genetics aspects, very little is known about possible disease-related protein changes, with the single exception of a proteomic study on a mouse model [8]. Among the several hundred RTT sporadic patients examined in the Child Neuropsychiatry Unit of the University Hospital of Siena, Italy, we have encountered two rare familial cases consisting of two pairs of sisters with RTT that are phenotypically discordant as previously reported [9]; that is, individuals in each pair demonstrate extremes of the RTT spectrum, that is, classical RTT and PSV-RTT. X chromosome inactivation (XCI) status is able to modulate X-linked disorders [10]. However, all four mentioned individuals show a balanced XCI, indicating that other factors beyond XCI may contribute to the phenotypic outcome [7, 11, 12]. Aim of the study was to unveil possible relationships between plasma proteome and phenotypic expression in two cases of familial RTT represented by two pair of sisters, harbor the same *MECP2* gene mutation while being dramatically discrepant in phenotype, that is, classical RTT versus PSV.

## 2. Materials and Methods

### 2.1. Subjects

**Figure 1:**
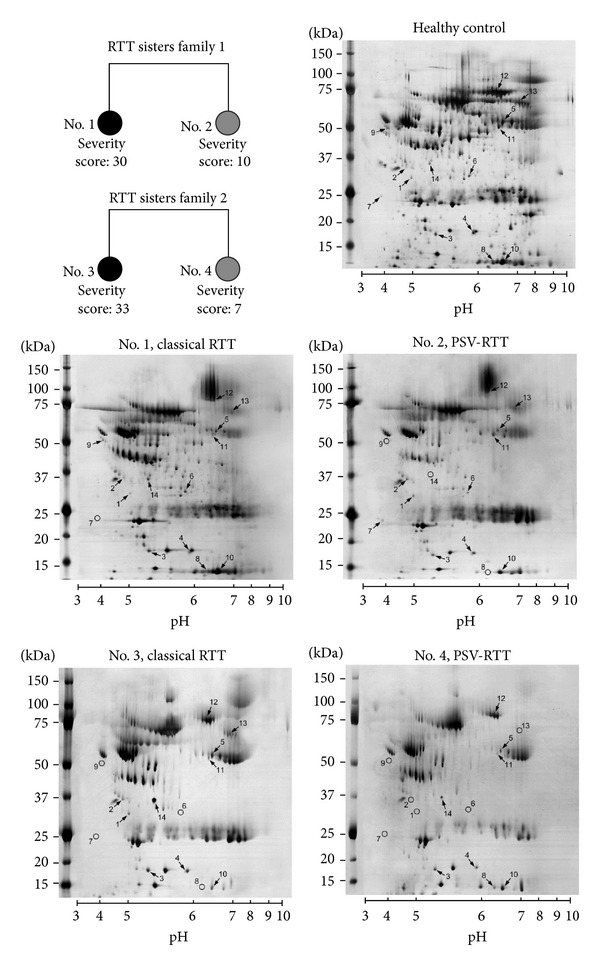
In the pedigrees the two RTT sisters families are represented by grey circles (milder variant = preserved speech variant, PSV-RTT) or black circles (more severe phenotype = classical RTT) with their respective clinical scores as derived by the approbation of phenotypical severity scale [9]. In the 2-DE maps typical control plasma proteome (healthy control), RTT sisters Family 1 (no. 1, no. 2) and RTT sisters Family 2 (no. 3, no. 4) are shown. Arrows indicate the protein spots with significant variations in their major or minor relative volume; circles are used to indicate the absence of the spots (i.e., qualitative variations).

Two pairs of sisters with discordant phenotype and identical mutation for each pair (pair 1: c.1157del32; pair 2: *de novo MECP2* deletion including exon 3 and part of exon 4) were enrolled in the present study [12]. Siblings no. 1 (42 years old) and no. 2 (33 years old) exhibits classical RTT and PSV-RTT, respectively. Both sisters showed a balanced XCI and inherited the same mutation from their unaffected mother, who had a completely skewed XCI [7]. Siblings no. 3 (34 years old) and no. 4 (40 years old) exhibits classical RTT and PSV-RTT, respectively. XCI status analysis in this couple of sisters revealed balanced XCI in both [12]. The unrelated classical RTT individuals no. 1 and no. 3 could not speak and walk and had a profound intellectual deficit, while the PSV individuals no. 2 and no. 4 could speak and walk and had a moderate intellectual disability (PSV-RTT). Striking differences in somatic, neurodevelopmental, and neurovegetative features between the sisters were present. A full clinical description of the affected siblings has been already reported by Grillo et al. [9]. The diagnostic criteria for the PSV form of RTT have been previously reported [13]. Mean classical RTT and PSV scores were, respectively, 27.5 ± 5.3 and 13.8 ± 5.9 (see the two pedigrees in Figure 1).

Gender and age-matched controls were also enrolled in the study. Blood samplings in the control group (*n* = 10) were carried out, during routine health checks or blood donations, always followed by written informed consent. This study was approved by the institutional review board of AOUS, Siena, Italy.

### 2.2. Blood Sampling

All samplings from RTT patients and healthy controls were carried out around 8 a.m. after overnight fasting. Blood was collected in heparinized tubes and all manipulations were carried out within 2 h after sample collection. The blood samples were centrifuged at 2400 g for 15 min at 4°C; the platelet poor plasma was saved and the buffy coat was removed by aspiration. Plasma samples were stored at −70°C until use.

### 2.3. 2-DE Analysis

2-DE was performed according to Görg et al. [14] with slight modifications. Samples containing 60 *μ*g of protein as determined by Bradford [15] were denatured with 10 mL of a solution containing 10% of sodium dodecyl sulfate (SDS), 2.3% of dithiothreitol (DTT) heated to 95°C for 5 min. The sample was then combined with 350 mL of solubilizing buffer containing 8 M urea, 2% of 3-[(3-cholamidopropyl)-dimethylammonio]-1-propane sulfonate (CHAPS), 0.3% DTT, 2% of immobilized pH gradient (IPG) buffer, and a trace of bromophenol blue and loaded into 18 cm IPG strips 3–10 NL on an Ettan IPGphor (GE Healthcare) apparatus system and rehydrated for 7 h. Isoelectric focusing (IEF) was carried out for a total of 32 kV h. After focusing, the strips were first equilibrated with equilibration buffer containing 50 mM Tris-HCl, pH 8.8, 6 M urea, 2% w/v SDS, 30% v/v glycerol, and 1% w/v DTT for 15 min; then they were equilibrated again with the same equilibration buffer described above, except that it contained 4% w/v iodoacetamide instead of DTT and a trace of bromophenol blue. The strips were washed further for 10 min with Tris-glycine buffer. The second dimension was performed on an EttanDalt Six Electrophoresis system (GE Healthcare). IPG strips and a molecular weight standard were embedded at the top of a 1.5 mm thick vertical polyacrylamide gradient gel (8–16% T) using 0.5% w/v agarose and run at a constant current of 40 mA/gel at 20°C. Each sample was carried out in triplicate under the same conditions. The exposure time for silver staining was also optimized to avoid overexposure of some gels with respect to others.

### 2.4. Tryptic Digestion and MALDI-TOF MS

After mass spectrometry compatible silver staining [28], the preparative gel was matched to the master gel in the analytical gel match set. A spot-picking list was generated and exported to Ettan Spot Picker (GE Healthcare). The spots were excised and delivered into 96-well microplates where they were destained and dehydrated with acetonitrile (ACN) for subsequent rehydration with trypsin solution. Tryptic digestion was carried out overnight at 37°C. Each protein spot digest (0.75 mL) was spotted into the MALDI instrument target and allowed to dry. Then 0.75 mL of the instrument matrix solution (saturated solution of *α*-cyano-4-hydroxycinnamic acid in 50% ACN and 0.5% v/v trifluoroacetic acid) was applied to dried samples and dried again. Mass spectra were obtained, as described [29], using an ultrafleXtreme MALDI-ToF/ToF (Bruker Corporation, Billerica, MA, USA).

### 2.5. Protein Identification by MS

After tryptic peptide mass acquisition, mass fingerprint searching was carried out in Swiss-Prot/TREMBL and NCBInr databases using MASCOT (Matrix Science, London, UK, http://www.matrixscience.com/). A mass tolerance of 100 ppm was allowed and only one missed cleavage site was accepted. Alkylation of cysteine by carbamidomethylation was assumed as a fixed modification, whereas oxidation of methionine was considered a possible modification. The criteria used to accept identifications included the extent of sequence coverage, number of matched peptides, and probabilistic score.

### 2.6. Image and Statistical Analysis

Images of gels were analyzed using ImageMaster 2D Platinum v7.0 software (GE Healthcare). The reference gel for each group (i.e., RTT, controls, classical RTT, PSV-RTT, RTT sisters Family 1, RTT sisters Family 2, and cases no. 1, no. 2, no. 3, and no. 4) was defined and used for the comparative analyses. Statistical analysis for protein differently expressed in the groups was carried out using GraphPad Prism software and the MedCalc version 12.1.4 statistical software package (MedCalc Software, Mariakerke, Belgium) was used. All variables were tested for normal distribution (D'Agostino-Pearson test). Data were expressed as median values and interquartile range, unless otherwise stated. Unmatched spots or spots with significantly different percentage volume (%V) were considered as “differently expressed”. Differences between groups were tested by the nonparametric Mann-Whitney rank sum test or Kruskal-Wallis analysis of variance, as appropriate. A two-sided *P* < 0.05 was considered to indicate statistical significance.

## 3. Results

To better characterize the RTT plasma protein pattern, we carried out a proteomic analysis based on 5 different analytical groups: (1) classical RTT versus PSV-RTT, (2) RTT versus controls, (3) RTT sisters Family 1 versus RTT sisters Family 2, (4) no. 1 classical RTT versus no. 3 classical RTT, and (5) no. 2 PSV-RTT versus no. 4 PSV-RTT. Among these groups there were significant quantitative and qualitative variations in 14 protein spots subsequently identified by mass spectrometry. Protein name as well as peptide matches, sequence coverage, and the probabilistic score obtained using the MASCOT software are summarized (Table 1). All the identified proteins are known to be involved in specific biological processes [16–27]. Proteomic plasma maps of healthy control, RTT sisters Family 1, and RTT sisters Family 2 with the protein spots are represented (Figure 1). Black arrows indicate the spots with quantitative variations while all the identified qualitative variations are reported with black circles.

**Figure 2:**
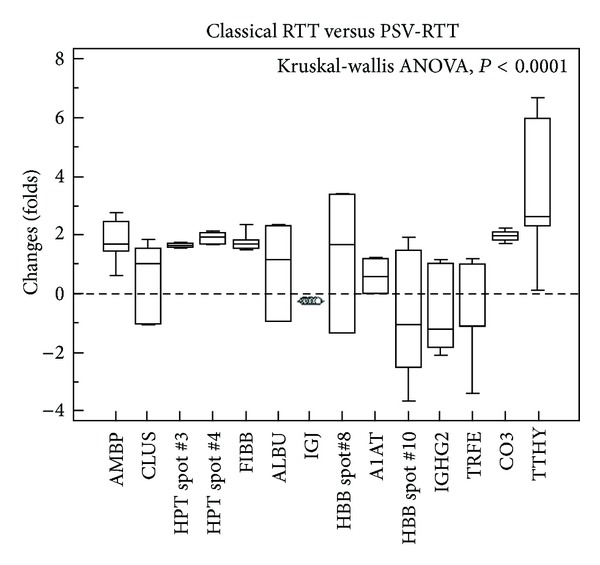
Plasma proteins expression in sisters with classical Rett syndrome as protein expression ratios of classical RTT versus PSV-RTT plasma proteome. Data are expressed as box-and-whiskers plots. Results of the Kruskal-Wallis ANOVA are shown.

As shown in Figure 2, significant changes appeared in alpha-1-microglobulin (AMBP), haptoglobin (HPT/Hp, spots 3 and 4), fibrinogen beta chain (FIBB), complement C3 (CO3), and transthyretin (TTHY) in classical RTT siblings as compared to PSV-RTT sisters. In addition, quantitative and qualitative protein variations values as derived from the examined RTT sister pairs and healthy controls comparative analyses were reported (Tables 2 and 3).

RTT patients, when compared to control group, showed 6 underexpressed protein spots including FIBB, hemoglobin subunit beta (HBB), serum transferrin (TRFE), HPT, Ig gamma-2 chain C region (IGHG2), and CO3, while 1 spot of clusterin (CLUS) is overexpressed (Table 3).

Family 1 versus Family 2 (third group in Table 3) showed a significant underexpression of TTHY, a significant overexpression of HBB, and the appearance of one protein spot of albumin (ALBU). The comparison between the two classical forms showed underexpression of 4 protein spots (AMBP, CLUS, IGHG2, and TTHY) and appearance of 3 protein spots (ALBU, HBB, and A1AT) in no. 1 as compared to no. 3. Significant qualitative variations are most evident in the comparison between the two PSV variants in which 5 protein spots appeared (AMBP, CLUS, ALBU, immunoglobulin J chain, IGJ and CO3) while 2 proteins (HBB and TTHY) disappeared in no. 2 as compared to no. 4. Moreover, in the same comparative group, an overexpression of HPT was observed (Table 3). In addition, another comparative analysis of each RTT patient versus healthy controls has resulted in several significant quantitative and qualitative variations in plasma proteome (see Table 4 in Supplementary Material available online at http://dx.doi.org/10.1155/2013/438653) and a number of protein spots changes likely due to the size effect originating from the healthy control group were detected but considered to be not significant (data not shown) and not comparable (*n.c.*, Table 2).

## 4. Discussion

Proteomic analysis has proven effective in identifying variations in proteins with biological and/or clinical significance [30]. The examined RTT siblings pairs represented an interesting benchmark model to test the molecular basis of phenotypical expression variability and to identify potential therapeutic targets of the disease.

Rett syndrome is the result of a monogenic mutation, that is, the X-linked *MECP2* gene in the overwhelming majority of cases. As RTT is an X-linked trait and the *MECP2* locus is subject to X inactivation, different patterns of X inactivation may lead to different phenotypes within a group of patients who carry the same mutation. Based on these data some authors speculate that there might be a group of RTT patients with milder phenotypes owing to skewed X inactivation, who have not so far been identified because of their atypical phenotypes. Nonetheless, variations in XCI are known to explain only 1/5 of the variance in severity of the disease [31], thus not fully accounting for the phenotype severity range typically seen in RTT [32].

We can safely state that in our patients, as well as in the majority of Rett syndrome patients reported in the literature, X inactivation was found to be balanced. Thus, it is reasonable to assume that the clinical phenotype of our pairs of sisters appears to be determined mainly by the type and location of the *MECP2* mutations.

Statistical analysis, represented by the fold changes, revealed in the classical RTT versus PSV-RTT comparison a significant overexpression of proteins involved in APR including AMBP, HPT, FIBB, A1AT, and CO3 [16, 18, 23, 26]. Although little is known about the APR and the frequency of infections in RTT [33], our findings evidenced a lack of some key APR components. Possible explanations for these findings may include a continuous stimulation of cytokine-mediated liver protein synthesis, an accelerated turnover of APR proteins, or a combination of both. The evidence that the RTT patients present chronic terminal bronchiolitis and an increase in intestinal microbiome due to constipation suggest the coexistence of recurrent infections [34, 35]. Evidence of the involvement of inflammatory events in RTT, was mainly represented by the significant variations of AMBP and A1AT (fifth and sixth comparative groups in Table 3), two serine protease inhibitors linked to the acute phase reaction, which limit the damage caused by activated neutrophils and their enzyme elastase [16, 23]. A major role for the immune system in RTT pathogenesis has been previously documented by the fact that transplantation of wild-type bone marrow restores wild-type microglia and arrests pathology in a mouse model of RTT [36].

The other finding on a partial deficit in an oxygen transport HBB [22] could be compatible with our prior finding of a subclinical hypoxia with an altered redox status in RTT patients with the classical phenotype [37].

Interestingly, TRFE was significantly underexpressed in RTT as compared to healthy controls, confirming the association previously reported in autism [38]. Alterations in the TRFE levels may lead to abnormal iron metabolism in RTT; it has been suggested for autism [38]. On the other hand, TRFE is also a negative APR protein whose expression levels decrease during inflammation [25]. Thus the underexpression of TRFE in RTT suggests once again that inflammatory process may play a key role in the pathogenesis of the disease.

More intriguing is the finding of an overexpressed CLUS, which may reflect a counterbalanced response to excessive proteins accumulation, namely, the unfolded protein response [17]. Abundant evidences demonstrated that CLUS expression is increased during cellular stress [17]. The chaperone action of CLUS could be cytoprotective in either or both the intra- or extracellular environments. In particular, extracellular CLUS binds to and prevents aggregation of partly unfolded proteins such as receptors on the surface of stressed cells [17]. This action may promote cell survival by minimizing stress-induced aberrant signaling.

Of course, our results have to be confirmed in a larger patients population. In the present study, we focused on the comparison between the two RTT variants by exposing the differences in protein patterns. Both siblings with classical RTT had a significant overexpression of HPT, one of the APR proteins induced in response to infection, tissue injury, and malignancy. HPT was originally described as functioning by the absorption of free hemoglobin and prevention of the consequent kidney damage [18]. However, it has subsequently became apparent that the physiological role of HPT is not limited to the trapping of free hemoglobin. Bacteriostatic and angiogenic effects, antibody-like and antioxidative properties have also been reported [18]. Evidence of an association between the Hp 2-2 phenotype and neurological disorders, like epilepsy and autism, has been reported [39, 40]. In support of this, we have distinguished Hp 2-2 phenotype in the 2-DE plasma maps corresponding to the two PSV-RTT (no. 2 and no. 4) and to a no. 3 classical RTT, referring to the previous 2-DE reference plasma maps reported in the literature [41]. Hp 2-2 phenotype is associated with a higher immune reactivity and ability to form antibodies. Moreover, the possession of this particular phenotype has been associated with the prevalence and clinical evolution of many inflammatory diseases including infections as tuberculosis, vaccination, viral hepatitis, atherosclerosis, and cardiovascular and autoimmune diseases [18, 39, 42]. Furthermore, Hp 2-2 shows lower binding of hemoglobin and antioxidant capabilities than Hp 1-1 phenotype [18].

Both classical RTT subjects also likely had a significant overexpression of FIBB which has a double function: yielding monomers that polymerize into fibrin and acting as a cofactor in platelet aggregation [19]. Moreover, FIBB expression level can be greatly increased as key component of the APR following tissue injury and infection/inflammation [19].

Our results also suggest that classical RTT subjects, given the significant variation observed in TTHY, may be likely more prone to have a dysfunction in thyroid hormone binding and transport proteins [27]. Physiologically, TTHY is responsible for thyroid hormone and retinol transport, through the binding of retinol binding protein [27]. Evidence of underexpression of TTHY and thyroid dysfunction has been reported in patients with RTT [43, 44].

Underexpression and/or disappearance of TTHY in the third, fourth, and fifth analytical groups (see Table 3) may suggest a possible relationship of this protein with oxidative stress (OS), as its changes could be related to low plasma retinol levels, in turn contributing to the production of reactive oxygen species [45]. The already solid evidence of enhanced OS and lipid peroxidation in RTT patients at different stages and with different gene mutations [37] seems to be in line with this interpretation.

We used plasma samples in order to embrace all the protein components of the blood soluble as the plasma proteome represents the largest and deepest version of the human proteome present in any sample [30].

Interestingly, in our findings plasma APR proteins (i.e., AMBP, HPT, FIBB, A1AT, and CO3) are five out of six (i.e., 83.3%) of the overexpressed proteins found in the classical sisters as compared with the PSV ones. TTHY might be the result of an adaptive endocrinological response to yet be clarified inflammatory stimuli. Therefore, inflammation could represent a potential novel target for the disease and inflammation-modulating drugs might be tested for the reduction of phenotype severity. To this regard, the naturally occurring and less aggressive anti-inflammatory molecules *ω*-3 polyunsaturated fatty acids (PUFAs) have been suggested to reduce phenotype severity in RTT [46–49]. Therefore, a proteomic analysis of plasma samples from RTT patients could provide a personalized pharmacological intervention. The study of sibling pairs, that is, partially genetically related subjects, further stresses the importance of personalizing the treatment. This kind of studies may contribute to a better understanding of the biological mechanisms for the observed benefits of *ω*-3 PUFAs supplementation in classical RTT patients. Actually, our unpublished data seem to suggest that *ω*-3 PUFAs supplementation is less efficient for PSV-RTT patients (J. Hayek, unpublished data).

Of note, our study may have identified novel targets for personalized RTT pharmacological intervention. To date there are no specific treatments to counterbalance protein expression and to reduce some of the clinical outcomes of RTT patients. Recently, a partial rescue of some of the neurological defects in RTT by *ω*-3 PUFAs has been reported [48]. Future plasma proteomics investigation on classical RTT and PSV-RTT patients treated with PUFAs would be an innovative strategy. This proteomic approach could be applied on patients presenting other clinical variants (ESV and congenital variant), in different tissues, cells, and biological fluids (i.e., cerebrospinal fluid, urine) and using experimental mouse and rat RTT models. Taken together, our results also suggest that (i) independently of the *MECP2* mutation type, some still unknown posttranscriptional modulating factors can be able to influence the clinical phenotype; (ii) these factors, combined with specific comutations [9], can determine alterations in the amount of plasma proteins (i.e., the significant increase of proteins involved in the inflammatory process, evident in the more severe phenotype) and/or hypothetically the functionality of some plasma proteins; (iii) there is a complexity degree high than previously thought as based on the exclusive effects of the *MECP2* gene mutation.

## 5. Conclusion

In summary, our results demonstrate that variations observed in RTT plasma proteome relate to proteins involved in several relevant biological processes other than those confined to the central nervous system. In particular APR/inflammation, blood coagulation, and OS response associated pathways appear to be involved. Our findings indicate that the study of unique familial cases offers the opportunity to identify new protein patterns involved in the RTT phenotype expression.

## Supplementary Material


*Additional Comparative Analysis*.
In this work, to better characterize the RTT plasma protein pattern, we carried out a proteomic analysis based on 5 different analytical groups: (1) classical RTT versus PSV-RTT, (2) RTT versus controls, (3) RTT sisters Family 1 versus RTT sisters Family 2, (4) no. 1 classical RTT versus no. 3 classical RTT, and (5) no. 2 PSV-RTT versus no. 4 PSV-RTT (see Table 3 in the main text).
In addition, to better understand the meaning of plasma proteome variations in each examined RTT patients, we carried out another 4 comparative analyses versus healthy controls. Significant quantitative variations appeared after these comparisons: No.1 classical RTT versus controls comparison showed an underexpression of alpha-1-microglobulin (AMBP), fibrinogen beta chain (FIBB), immunoglobulin gamma-2 chain C region (IGHG2), serum transferrin (TRFE) and complement C3 (CO3); No. 2 classical RTT versus controls comparison showed an underexpression of FIBB, albumin (ALBU), TRFE and CO3 and an overexpression of clusterin (CLUS); No. 3 PSV-RTT versus controls showed underexpressions of hemoglobin subunit beta (HBB), IGHG2, TRFE, CO3 and an underexpression of transthyretin (TTHY); No. 4 PSV-RTT versus controls showed underexpression of IGHG2 and TRFE. Significant qualitative variations appeared after these comparisons as protein disappearances: No.1 classical RTT versus controls comparison showed a disappearance of immunoglobulin J chain (IGJ); No. 2 classical RTT versus controls comparison showed disappearances of HBB, alpha-1-antitrypsin (A1AT) and TTHY; No. 3 PSV-RTT versus controls showed disappearances of ALBU, IGJ, HBB and A1AT; No. 4 PSV-RTT versus controls showed disappearances of AMBP, CLUS, ALBU, IGJ, A1AT and CO3 (Table 4).

## Figures and Tables

**Table 1:** Identification of plasma proteins in RTT patients and healthy controls by MS.

ID	AC^a^	Protein name	Short name	pI/Mr (kDa) predicted	pI/Mr (kDa) experimental	Peptide matches	Sequence coverage (%)	MOWSE score	Biological process involved; molecular function; references
1	P02760	Alpha-1-microglobulin	AMBP	5.07/30.9	5/31.1	9/15	25	77	Host-virus interaction; trypsin and plasmin inhibitor; [16]
2	P10909	Clusterin	CLUS	4.9/36.9	4.8/36.4	12/18	25	146	Chaperone; prevents stress-induced aggregation of blood plasma proteins; [17]
3	P00738	Haptoglobin	HPT	5.4/16.8	5.2/17	6/14	19	75	Immunity; captures hemoglobin, antimicrobial, and antioxidant; [18]
4	P00738	Haptoglobin	HPT	6.07/16.8	5.9/17	6/13	19	73	Immunity; captures hemoglobin, antimicrobial, and antioxidant; [18]
5	P02675	Fibrinogen beta chain	FIBB	6.4/55.2	6.6/55.2	37/88	60	231	Blood coagulation and hemostasis; [19]
6	P02768	Serum albumin	ALBU	5.6/67.7	5.8/68	5/7	8	56	Regulation of the osmotic blood pressure; binds ions, hormones, and fatty acids; [20]
7	P01591	Immunoglobulin J chain	IGJ	4.5/23.4	4.5/24	5/18	32	61	Immunity; links two monomer units of either IgM or IgA; [21]
8	P68871	Hemoglobin subunit beta	HBB	6.8/10.5	6.4/12.5	6/9	53	110	Oxygen transport; [22]
9	P01009	Alpha-1-antitrypsin	A1AT	4.8/50.3	4.8/50.2	10/14	32	141	Serine proteases inhibitor; [23]
10	P68871	Hemoglobin subunit beta	HBB	7.05/10.5	6.8/12.5	15/31	95	220	Oxygen transport; [22]
11	P01859	Ig gamma-2 chain C region	IGHG2	6.1/24.4	6/24.6	7/40	17	44	Immunity; antigen binding; [24]
12	P02787	Serum transferrin	TRFE	6.3/80.7	6.3/79.3	36/71	45	311	Iron binding transport proteins which can bind two Fe^3+^ ions; [25]
13	P01024	Complement C3	CO3	6.6/70.6	6.8/69.7	16/21	14	144	Immunity; central role in the activation of the complement system; [26]
14	P02766	Transthyretin	TTHY	5.5/35.3	5.4/34.4	9/27	77	136	Thyroid hormone-binding protein; [27]

Spot ID refers to that shown in 2-DE maps (Figure 1). ^a^Accession numbers of Swiss-Prot or GenBanK databases.

**Table 2:** Quantitative and qualitative protein variations values.

ID	AC^a^	Short name	Classical versus PSV		RTT versus healthy controls		Family 1 (*F1*) versus Family 2 (*F2*)		No. 1 Classical versus no. 3 Classical		No. 2 PSV versus no. 4 PSV	
			No. 2, no. 4	No. 1, no. 3	Controls	*F1, F2 *	*F2 *	*F1 *	No. 3	No. 1	No. 4	No. 2
1	P02760	AMBP	0.63 ± 0.69	1.29 ± 0.35	1.92 ± 0.36	0.96 ± 0.63	0.80 ± 0.88	1.12 ± 0.18	1.59 ± 0.12	0.99 ± 0.12*	n.d.	1.26 ± 0.11
2	P10909	CLUS	0.93 ± 1.02	1.21 ± 0.34	0.51 ± 0.23	1.07 ± 0.74**	0.75 ± 0.83	1.39 ± 0.52	1.50 ± 0.17	0.92 ± 0.05*	n.d.	1.87 ± 0.08
3	P00738	HPT	2.14 ± 0.31	3.56 ± 0.19**	3.56 ± 1.04	2.85 ± 0.78	2.98 ± 0.65	2.72 ± 0.94	3.56 ± 0.16	3.57 ± 0.25	2.40 ± 0.09	1.88 ± 0.18
4	P00738	HPT	2.53 ± 0.80	4.88 ± 0.62**	5.74 ± 1.49	3.70 ± 1.40**	3.08 ± 1.41	4.33 ± 1.18	4.35 ± 0.34	5.40 ± 0.08	1.81 ± 0.03	3.26 ± 0.11**
5	P02675	FIBB	2.02 ± 0.26	3.65 ± 0.63**	7.35 ± 3.10	2.83 ± 0.97**	3.01 ± 1.26	2.66 ± 0.62	4.11 ± 0.56	3.18 ± 0.20	1.90 ± 0.16	2.13 ± 0.33
6	P02768	ALBU	0.94 ± 1.02	1.08 ± 1.19	3.49 ± 1.34	1.01 ± 1.06	n.d.	2.02 ± 0.17	n.d.	2.17 ± 0.09	n.d.	1.87 ± 0.04
7	P01591	IGJ	0.22 ± 0.24	n.d.	0.55 ± 0.16	0.11 ± 0.19	n.d.	0.22 ± 0.24	n.d.	n.d.	n.d.	0.43 ± 0.02
8	P68871	HBB	1.34 ± 1.47	2.25 ± 2.46	4.81 ± 2.37	1.80 ± 1.99	1.34 ± 1.47	2.25 ± 2.46	n.d.	4.50 ± 0.04	2.68 ± 0.07	n.d.
9	P01009	A1AT	n.d.	0.60 ± 0.26	2.57 ± 1.24	0.30 ± 0.54	n.d.	0.60 ± 0.26	n.d.	1.20 ± 0.03	n.d.	n.d.
10	P68871	HBB	6.91 ± 2.61	6.59 ± 4.53	10.38 ± 4.30	6.75 ± 3.53*	3.85 ± 1.50	9.65 ± 2.25**	2.93 ± 1.07	10.26 ± 3.15	4.78 ± 1.37	9.03 ± 1.25
11	P01859	IGHG2	1.97 ± 1.56	1.61 ± 0.57	4.16 ± 1.57	1.79 ± 1.14**	1.34 ± 0.86	2.25 ± 1.27	2.12 ± 0.15	1.11 ± 0.18**	0.56 ± 0.09	3.39 ± 0.35
12	P02787	TRFE	3.21 ± 0.44	3.11 ± 0.50	10.51 ± 3.90	3.16 ± 0.45**	3.30 ± 0.61	3.03 ± 0.18	3.26 ± 0.72	2.96 ± 0.22	3.33 ± 0.65	3.09 ± 0.15
13	P01024	CO3	0.99 ± 1.08	2.04 ± 0.31	4.64 ± 1.32	1.51 ± 0.94**	1.12 ± 1.24	1.91 ± 0.12	2.23 ± 0.34	1.85 ± 0.15	n.d.	1.97 ± 0.06
14	P02766	TTHY	1.34 ± 1.47	5.86 ± 2.91**	2.61 ± 0.83	3.60 ± 3.20	5.59 ± 3.20	1.61 ± 1.78**	8.49 ± 0.56	3.23 ± 0.30**	2.69 ± 0.04	n.d.

Spot ID refers to that shown in 2-DE maps (Figure 1). ^a^Accession numbers of Swiss-Prot or GenBanK databases. Proteins values are expressed as relative %V (mean ± SD). For proteins significantly decreased or increased: *P < 0.05, **P < 0.01. n.d.: not detected.

**Table 3:** Protein variations as derived from the examined RTT sister pairs and healthy controls comparative analyses.

Analytical groups	Plasma proteome differences			
	Quantitative variations		Qualitative variations	
	Underexpressed	Overexpressed	Disappearance	Appearance
Classical RTT^(2)^ versus PSV-RTT^(2)^	N.D.	↑ AMBP*, ↑ HPT*, ↑ FIBB*, ↑ CO3*, ↑ TTHY*	N.D.	A1AT
RTT^(4)^ versus controls^(10)^	↓ HPT, ↓ FIBB, ↓ HBB, ↓ IGHG2, ↓ TRFE, ↓ CO3	↑ CLUS	N.D.	N.D.
RTT sisters Family 1^(2)^ versus RTT sisters Family 2^(2)^	↓ TTHY	↑ HBB	N.D.	+ALBU
No. 1 classical RTT^(1)^ versus no. 3 classical RTT^(1)^	↓ AMBP, ↓ CLUS, ↓ IGHG2, ↓ TTHY	N.D.	N.D.	+ALBU, +HBB, +A1AT
No. 2 PSV-RTT^(1)^ versus no. 4 PSV-RTT^(1)^	N.D.	↑ HPT	−HBB, −TTHY	+AMBP, +CLU, +ALBU, +IGJ, +CO3

↓: protein spot underexpressed; ↑: protein spot overexpressed; −: protein spot disappearance; +: protein spot appearance; N.D.: not detectable; *changes referred to relative variations between classical RTT and PSV-RTT siblings.

A1AT: alpha-1-antitrypsin; AMBP: alpha-1-microglobulin; ALBU: albumin; CLUS: clusterin; CO3: complement C3; FIBB: fibrinogen beta chain; HBB: hemoglobin subunit beta; HPT: haptoglobin; IGHG2: immunoglobulin gamma-2 chain C region; IGJ, immunoglobulin J chain; TRFE, serum transferrin; TTHY: transthyretin. Numbers in the parentheses indicate the number of patients or subjects compared.
