# Supplementary material for: A Plasma Proteomic Approach in Rett Syndrome: Classical versus Preserved Speech Variant
Source: Mediators Inflamm. 2013 Dec 23;2013:438653. doi: 10.1155/2013/438653 (PMC3884802; doi:10.1155/2013/438653)
Supplement: Supplementary file 1 — Additional Comparative Analysis. In this work, to better characterize the RTT plasma protein pattern, we carried out a proteomic analysis based on 5 different analytical groups: (1) classical RTT versus PSV-RTT, (2) RTT versus controls, (3) RTT sisters Family 1 versus RTT sisters Family 2, (4) no. 1 classical RTT versus no. 3 classical RTT, and (5) no. 2 PSV-RTT versus no. 4 PSV-RTT (see Table 3 in the main text). In addition, to better understand the meaning of plasma proteome variations in each examined RTT patients, we carried out another 4 comparative analyses versus healthy controls. Significant quantitative variations appeared after these comparisons: No.1 classical RTT versus controls comparison showed an underexpression of alpha-1-microglobulin (AMBP), fibrinogen beta chain (FIBB), immunoglobulin gamma-2 chain C region (IGHG2), serum transferrin (TRFE) and complement C3 (CO3); No. 2 classical RTT versus controls comparison showed an underexpression of FIBB, albumin (ALBU), TRFE and CO3 and an overexpression of clusterin (CLUS); No. 3 PSV-RTT versus controls showed underexpressions of hemoglobin subunit beta (HBB), IGHG2, TRFE, CO3 and an underexpression of transthyretin (TTHY); No. 4 PSV-RTT versus controls showed underexpression of IGHG2 and TRFE. Significant qualitative variations appeared after these comparisons as protein disappearances: No.1 classical RTT versus controls comparison showed a disappearance of immunoglobulin J chain (IGJ); No. 2 classical RTT versus controls comparison showed disappearances of HBB, alpha-1-antitrypsin (A1AT) and TTHY; No. 3 PSV-RTT versus controls showed disappearances of ALBU, IGJ, HBB and A1AT; No. 4 PSV-RTT versus controls showed disappearances of AMBP, CLUS, ALBU, IGJ, A1AT and CO3 (Table 4). [file 438653.f1.pdf]

Table 4: Protein variations as derived from each examined RTT sister *vs*. healthy controls comparative analyses.

Comparative groups	Plasma proteome differences			
	Quantitative variations		Qualitative variations	
	Decrease	Increase	Disappearance	Appearance
#1 Classical RTT(1) *vs* Controls(10)	↓AMBP, ↓FIBB, ↓IGHG2, ↓TRFE, ↓CO3	N.D.	-IGJ	N.D.
#2 Classical RTT(1) *vs* Controls(10)	↓FIBB, ↓ALBU, ↓TRFE, ↓CO3	↑CLUS	-HBB, -A1AT, -TTHY	N.D.
#3 PSV-RTT(1) *vs* Controls(10)	↓HBB, ↓IGHG2, ↓TRFE, ↓CO3	↑TTHY	-ALBU, -IGJ, -HBB, -A1AT	N.D.
#4 PSV-RTT(1) *vs* Controls(10)	↓IGHG2, ↓TRFE	N.D.	-AMBP, -CLUS, -ALBU, -IGJ, -A1AT, -CO3	N.D.

↓ protein decrease; ↑ protein increase; - protein disappear; + protein appear; N.D. protein not detectable.

A1AT, Alpha-1-antitrypsin; AMBP, Alpha-1-microglobulin; ALBU, Albumin; CLUS, Clusterin; CO3, Complement C3; FIBB, Fibrinogen beta-chain; HBB, Hemoglobin subunit beta; HPT, Haptoglobin; IGHG2, Immunoglobulin gamma-2 chain C region; IGJ, Immunoglobulin J chain; TRFE, Serum transferrin; TTHY, Transthyretin. Numbers in the parentheses indicated the number of patients or subjects who are compared.
